# Sports participation related to injuries and illnesses among ambulatory youth with chronic diseases: results of the health in adapted youth sports study

**DOI:** 10.1186/s13102-019-0145-5

**Published:** 2019-12-16

**Authors:** Kristel Lankhorst, Janke de Groot, Tim Takken, Frank Backx, on behalf of the HAYS study group, F. J. G. Backx, J. F. de Groot, K. M. Lankhorst, T. C. W. Nijboer, T. Takken, D. W. Smits, O. W. Verschuren, J. M. A. Visser-Meily, M. J. Volman, H. W. Wittink

**Affiliations:** 1 Research Group Lifestyle and Health, Institute of Human Movement Studies, University of Applied Sciences, P.O. Box 85083, 3508AB, Utrecht, The Netherlands; 2 Department of Rehabilitation, Physical Therapy Science & Sports, Brain Center, University Medical Center Utrecht, Utrecht, the Netherlands; 3 Child Development and Exercise Center, Wilhelmina Children’s Hospital, University Medical Center Utrecht, Utrecht, the Netherlands; 4 Netherlands Institute for Healthcare Services Research (NIVEL), Utrecht, the Netherlands

**Keywords:** Injury, Illness, Youth, Disability, Sports participation, Chronic disease, Ambulatory

## Abstract

**Background:**

Although sports participation leads to important health enhancement for youth with chronic diseases or physical disabilities (CDPD), it may pose an increased risk for injury or illness. This study investigated the incidence, type, severity and risks to (sports-related) injuries and illnesses among ambulatory youth with CDPD.

**Methods:**

Over a 12-month period, every 2 weeks, the characteristics of injuries and illnesses were registered by an online questionnaire and phone-based interview. Physical activity level was measured with the Activ8 during 1 week. Complete data was available of 103 youngsters with CDPD (61 boys, 42 girls), with a mean age of 14.4 (SD = 2.7) years. The personal characteristics, the injury and illness rates per 1000 h of PA were investigated per group of organized sports participation per week (0, 1 or ≥ 2 times p/wk).

**Results:**

Almost half of the youngsters sustained one or more injuries (46%) or illnesses (42%) during 1 year. The injury rate per 1000 h of PA between 0, 1 and ≥ 2 times per week of sports participation was 0.84, 1.88, 133 respectively and the illness rate were 1.87, 1.88 and 1.18 respectively. Differences between the rates were not statically significant. Most reported health problems had no subsequent restriction (49%) or other minor consequences (21%) in school, physical education or sports participation. Most reported health problems were contusions (41%) at the lower extremity (74%) and flu plus fever (58%).

**Conclusions:**

Participation in sports ≥2 times per week does not pose a significant increased risk in the incidence of injury or illness per 1000 h of PA in youth with CDPD compared to their peers who participate less frequent (once weekly) and compared to non-sports participants. Athletes who participate in sports at least twice weekly get injured mostly during their sporting activities, while peers who do participate in sports once a week or not at all, get injured during less intense physical activities during physical eduction lessons, ADL or non-organized sports and play in leisure time. The social impact of injuries or illnesses was limited.

## Background

Sports participation has important health benefits in both healthy youth and peers with chronic diseases or physical disabilities (CDPD) [1–5]. Studies have shown strong associations between being member of a sports club and the amount of moderate-vigorous physical activity (MVPA) and vigorous activity levels (VPA) in youth with CDPD; those who are sport club members are twice as likely as non-members to meet international physical activity (PA) recommendations [5–7]. Meeting adequate PA levels supports a healthier lifestyle. In addition, sports participation also has major positive health effects. It contributes to a better fitness, a higher degree of PA, a higher quality of life, a better self-image and children find themselves more athletic skilled [3].

At the same time, sports participation is also known to lead to sports-related injuries and illnesses, as shown in adults, healthy youth and among youth with CDPD [5–7]. Sports injuries among healthy youth have a considerable impact on their participation and performance in subsequent activities [6, 8]. A longer existing injury or illness can limit participation in sports or lead to dropping out of sports or fear to return to sports among healthy youth [9]. Moreover, research among healthy youth shows that a low level of PA entails a higher risk of being injured when they become more active, while there is only limited evidence regarding sports participation and injuries in youth with CDPD [10, 11]. For youngsters with CDPD it is already a challenge to reach adequate levels of PA due to the existing social and environmental barriers for sports and exercise participation [12]. It is even harder to pursue an active and healthy lifestyle through participation in sports, when sports participation is associated with (fear of) injuries and/or illnesses. In addition, there is a reasonable fear among parents of children with CDPD that sports participation undoubtedly leads to sport-related injuries. As a consequence of that injury, their child could experience additional limitations in their daily lives, i.e. being unable to perform their daily activities independently anymore or needing more help with it from their parents or caregivers [13]. These negative experiences and beliefs with regard to sports participation can further limit the support to allow their child to be active in sports. In addition, a previous systematic review among adult athletes with disabilities recommends longitudinal and prospective cohort studies to gain more understanding and insight into factors influencing injuries and illnesses [14–17].

Therefore the aim of this study was to investigate the incidence, type, severity and risks of (sports-related) injuries and illnesses among ambulatory youth with CDPD over a 12-month period. We also investigated whether the more serious sports participants run a higher risk of being injured or ill compared to the less serious peers.

## Methods

### Study aim

Firstly, the aim of this study was to investigate the incidence, type, severity and risks of (sports-related) injuries and illnesses among ambulatory youth with CDPD over a 12-month period. Secondly, we investigated whether the more serious sports participants run a higher risk of being injured or ill compared to the less serious peers.

### Study design

The present study is part of the Health in Adapted Youth Sports (HAYS) Study, which involved an analysis of data about health-related fitness, PA and psychosocial health in youth with CDPD [2, 3, 18, 19]. This study was approved by the Medical Ethics Committee of the University Medical Center Utrecht, Utrecht, the Netherlands (METC number: 14–332/c). The present sub-study is a prospective cohort study specifically evaluating injuries and illnesses related to sports participation. The procedures and protocols of the HAYS Study have been published previously in more detail [18].

This study has two objectives; 1) to determine the incidence, type and severity of injuries or illnesses, 2) to calculate the injury and illness rates per 1000 h of PA per group among ambulatory youth with CDPD participating in organized sports once or twice a week and peers who do not participate in sports at all. These objectives are researched by means of an injury and illness registration system over a 12 months period among youth with CDPD. This prospective cohort study followed Strengthening the Reporting of Observational studies in Epidemiology (STROBE) guidelines [20].

### Participants

Ambulatory youth with CDPD were recruited for participation in the HAYS study between October 2014 and October 2016. The follow-up period and data collection of injuries and illnesses lasted till October 2017. The children and adolescents were recruited in the Netherlands among different patients associations, pediatric therapy practices, Wilhelmina Children’s Hospital in Utrecht, De Hoogstraat Rehabilitation Center in Utrecht, Fitkids network, schools for special education for children with a disability, and sports clubs. Young athletes were recruited from a broad range of participation in sports: from recreational level to high level competitive sports.

Participants were eligible for this study when they were ambulatory, aged from 8 to 19 years, and diagnosed with one or more cardiovascular, pulmonary, musculoskeletal, metabolic or neuromuscular disorders according to the classification of the American College of Sports Medicine [21].

Informed consent was provided by all participants and by the parents of participants under 18 years of age. In line with Dutch law, no parental informed consent was required for participants aged 18 years and above.

**Fig. 1:**
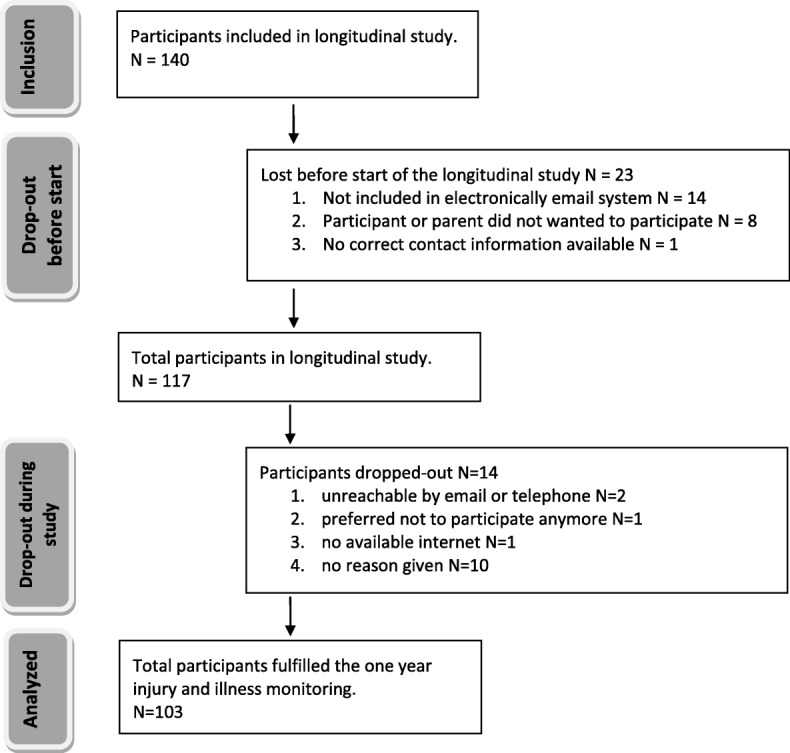
Number of participants included and dropped-out in the prospective cohort study. Brief description: the flowchart includes information about the number of participants included in the study by baseline, number of participants dropped out before the start and during the study, and total number of participants’ data that was analyzed

The HAYS cross-sectional sample involved 140 participants with CDPD, with a mean age (SD) of 14.4 (2.7) years (Table 1 and Fig. 1).

**Table 1:** Characteristics of the participants per (sports)-group

Sports participation per week	Group 0 (Non-sports)	Group 1 (sports 1x/wk)	Group 2 (sports ≥2x/wk)	Total	*p*-value
Number of participants	18	21	64	103	
Number of boys (%)^a^	9 (50)	8 (38)	44 (69)	61 (59)	0.032^c^
Age in years (SD) ^b^	15.4 (2.7)	14.2 (2.8)	14.1 (2.7)	14.4 (2.7)	0.219
Height in cm (SD) ^b^	165.3 (9.5)	159.2 (13.2)	161.2 (14.7)	161.9 (12.5)	0.361
Weight in kg (SD) ^b^	58.2 (13.5)	50.7 (16.4)	53.7 (17.6)	54.2 (15.8)	0.385
BMI (SD) ^b^	21.1 (3.7)	19.7 (4.6)	20.2 (3.9)	20.2 (4.1)	0.546
BMI – age SDS (SD) ^b^	0.59 (1.2)	0.11 (1.9)	0.51 (1.2)	0.44 (1.4)	0.449
Medical diagnosis (%)^a^					0.411
*- Cardiovascular disease*	3 (17)	4 (19)	4 (6)	11 (11)	
*- Pulmonary disease*	0	2 (9)	5 (8)	7 (7)	
*- Metabolic disease*	1 (6)	1 (5)	6 (9)	8 (8)	
*- Musculoskeletal / orthopedic disability*	1 (6)	1 (5)	6 (9)	8 (8)	
*- Neuromuscular disease / disability*	6 (33)	9 (43)	31 (49)	46 (46)	
*- Immunological / hematological disease*	5 (28)	4 (19)	6 (9)	15 (15)	
*- Cancer*	0	0	1 (2)	1 (1)	
*- Epilepsy*	2 (10)	0	5 (8)	7 (7)	

*BMI* body mass index, *SD* standard deviation, *SDS* standard deviation score,^a^ chi-square test for sex and prevalence of diseases or disabilities per group. ^b^ ANOVA for age, length, weight, BMI and BMI-age SDS. ^c^ Significant difference

### Procedure and data collection

**Fig. 2:**
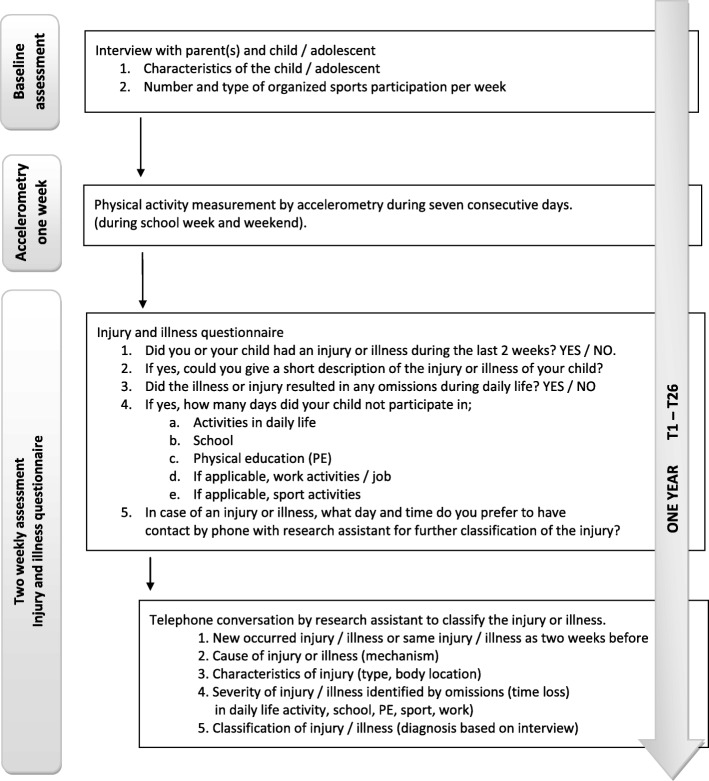
Online questionnaire and phone based assessment of injury or illness. Brief description: the flowchart includes information about the number of participants per measurement; baseline assessment, accelerometry during 1 week and follow-up assessment during a 12-month period

An overview of the procedures followed and data collection of the study are summarized in Fig. 2. Data collection consisted of: 1) a baseline assessment with the child or adolescent and parent(s); 2) objectively measured PA level of the participants during one school week; and 3) a two weekly registration of each injury or illness, for 12 months following the baseline assessment.

The characteristics of the child or adolescent were collected by the baseline assessment, including age, sex, medical diagnosis, participation in physical education (PE) and the number and type of sports participation in organized sports per week. Sports participation at baseline was identified using the following three standardized questions used by the Dutch Institute of Health and Environment [22]: 1) do you participate in sports?; 2) what is/are the type of organized sport(s)?; and 3) what is the frequency of participation in organized sports per week?.

In this study, participants were classified by the frequency of participation in organized sports per week (during a normal school week) to gain insight into sports-related injuries, non-sports-related injuries i.e. injuries related to PA during leisure time. This resulted in the following three groups: group 0 = no participation in organized sport at all; group 1 = sports participation at sport club one time per week, group 2 = sports participation at sport club two or more times per week.

Exposure to sport/PA was measured using an activity monitor, the Activ8 (2 M Engineering Ltd. Valkenswaard, The Netherlands). The Activ8 is a valid one-sensor ambulatory monitoring system and has been validated for use in youth with and without motor impairments [23]. Each subject wore the sensor on the dominant leg, fixed with Tegaderm™ (3 M, Delft, the Netherlands) waterproof skin tape during seven consecutive days for 24 h each day. The type, duration and frequency of PA in daily life were measured during a school week and one weekend. In order to calculate and interpret the data of waking hours gathered with the Activ8, also sleeping time was recorded in a diary. The total active time in minutes per day during leisure time, PE and organized sports per week was used for calculations. The analysis and results of PA data in the HAYS study are described elsewhere in more detail [18].

The monitoring of injuries and/or illnesses during the 12-month follow-up period was conducted by use of an online questionnaire. This questionnaire was developed based on recommendations of the Dutch Ministry of Health, Welfare and Sport (VWS) [24] and designed in Formdesk, an online web-based tool (Innovero Sofware Solutions B.V., Wassenaar, The Netherlands). Following the baseline assessment, the participants and/or their parents received an email with a hyperlink to the online questionnaire every 2 weeks for 12 months (Fig. 2). The questionnaire included five simple questions concerning injuries and/or illnesses suffered during the past 2 weeks, resulting in 26 measurements per participant. The participants received a reminder by e-mail if the questionnaire was not completed within 3 days. After three more days, the research assistant contacted the participant by telephone when the questionnaire was not completed, preventing an incomplete dataset. We performed a structured interview by phone in case of an injury or illness reported in the online questionnaire. In the personal interview we registered the type and body location of the injury or type of illness and the severity of the injury or illness.

### Definitions of injury and illness

The injury definition according to the National Athletic Injury Registration System (NAIRS) [17] was adapted for use in this study as follows:

*‘Any new musculoskeletal pain, feeling or injury which results from participation in recreational PA or sports and causes changes in physical activities including sports activities, regardless of whether or not time is lost from PA, sports training or competition.’*

An illness was defined and adapted for use in this study as:

*‘Any new illness that causes changes in physical activities including sports activities, regardless of whether or not time is lost from PA or sports training or competition.’* [25]

Injuries and illnesses were further classified by the number of days of participation restriction in school and PE, in agreement with previous injury surveillance research [26] and according to the NAIRS [17]. Reported injuries or illnesses were classified as non-time loss (NTL) injuries or illnesses when the injury or illness complaint only had a participation limitation on the same day of the injury or illness complaint and not the day after [26]. Reported injuries or illnesses were classified as time-loss (TL) injuries or illnesses when there was a participation restriction of at least 1 day after the day of onset of the injury or illness complaint. To indicate the severity of the TL injuries and illnesses, the TL injury or illness were further subcategorized as follows: minor (1–7 days lost), moderate (8–21 days lost) or severe (> 21 days) [17, 26].

### Primary outcome – absolute incidence, characteristics and severity of injuries and illnesses

The data regarding the occurrence of injuries (body location and type) and illnesses (type) and consequences (time-loss in days until return to school, PE and participation in sports) were collected for all three groups.

### Secondary outcome - injury and illness rates per 1000 h PA

The injury and illness rates per 1000 h of PA was calculated for each group (group 0, 1 and 2). An injury or illness rate indicates how many injuries or illnesses occur per episodes of exposure to sport and/or PA.

The injury and illness rate is calculated by the following formula:

Injury or illness rate =

*(the number of injuries or illnesses during 12-months /*

*the total hours exposure to PA during 12-months) × 1000.*

## Statistical analysis

Descriptive analyses were used to profile characteristics of the three groups. An ANOVA test was used determining the differences between the groups for age, length, weight, BMI and BMI-age standard deviation score (SDS). The chi-square test was used determining the differences between the three groups for sex and prevalence of diseases or disabilities. The dependent variable is the occurrence of injuries and illnesses within a 12-month period and the amount of organized sports participation per week as independent variables. We also investigated whether the more serious sports participants (two or more times of sports participation p/wk.) run a higher risk of being injured or ill compared to the less serious peers who participate in sports once per week (group 1) and not at all (group 0). Group 1 and 0 were taken together as one group for this additional analysis. Analyses were performed using the SPSS Statistics, Version 23.0 (IBM Corp., Armonk, NY, USA). Determining the differences between the three groups for the incidence of injuries and illnesses (injury and illness rates) during the 12-month follow-up period and calculation of the 95% confidence intervals for injury and illness rates were performed using MedCalc for Windows, version 17.9 (MedCalc Software, Ostend, Belgium). A *p* value < 0.05 was considered statistically significant.

## Results

### Participants’ characteristics

Of 140 children and adolescents with CDPD who were invited to participate, 103 completed the 12 month period of prospective injuries and illnesses monitoring and only their data were analyzed for the current study. Reasons for dropout during the 12 month period of prospective monitoring are presented in Fig. 1. We lost 23 (16%) participants before the start of the study mainly caused by faults in our email system or participants/parents did not want to participate. Further, fourteen participants (10%) dropped out during the follow-up period of the study. The drop out of participants was not related to personal characteristics (i.e. diagnosis, age, sex) and was randomly between the three sub-groups.

The participants’ characteristics (sex, age, medical diagnosis, height, weight, BMI and BMI SDS) and *p*-value per group are displayed in Table 1.

The vast majority of the group had a neuromuscular disease (46 out of 103), with Gross Motor Function Classification System (GMFCS) classification of 1 or 2. There was a significant difference between the number of participants per group and for sex in group 2, 44 boys versus 20 girls (*p*-value = 0.031) Table 2.

**Table 2:** Incidence and severity of injuries and illnesses categorized according return to play (PE or sports) per group over a 12-month period

Sports participation per week	Group 0 (Non-sports)	Group 1 (Sports 1x/wk)	Group 2 (Sports ≥2x/wk)	Total (%)
Number of participants	18	21	64	103
INJURIES				
Number of participants with an injury (%)	5 (28)	7 (33)	35 (55)	47 (46)
Total number of injuries registered (%)	9 (10)	17 (20)	60 (70)	86
*Severity of injuries with restriction for return to school*^*a*^ *(N = 53)*				
No time loss (NTL) (%)	3 (33)	6 (35)	33 (55)	42 (49)
Minor (1–7 days) (%)	1 (11)	2 (12)	6 (10)	9 (10)
Moderate (8–21 days) (%)	0	0	1 (2)	1 (1)
Severe (> 21 days) (%)	0	0	1 (2)	1 (1)
*Severity of illnesses for return to PE / Sports*^*a*^ *(N = 53)*				
No time loss (NTL) (%)	8 (89)	10 (59)	24 (40)	42 (49)
Minor (1–7 days) (%)	0	3 (18)	15 (25)	18 (21)
Moderate (8–21 days) (%)	0	1 (6)	13 (22)	14 (16)
Severe (> 21 days) (%)	1 (11)	3 (17)	8 (13)	12 (14)
ILLNESSES				
Number of participants with illnesses (%)	9 (50)	9 (43)	25 (39)	43 (42)
Total number of illnesses registered (%)	20; range 1–7 (22)	17; range 1–3 (19)	53 range; 1–6 (59)	90
*Severity of illnesses with restriction for return to school*^*a*^ *(N = 90)*				
No time loss (NTL) (%)	5 (25)	2 (12)	10 (19)	17 (20)
Minor (1–7 days) (%)	12 (60)	12 (71)	31 (59)	55 (64)
Moderate (8–21 days) (%)	1 (5)	3 (18)	6 (11)	10 (12)
Severe (> 21 days) (%)	2 (10)	0	6 (11)	8 (9)
*Severity of illnesses for return to PE / Sports*^*a*^ *(N = 90)*				
No time loss (NTL) (%)	15 (75)	10 (59)	9 (17)	34 (40)
Minor (1–7 days) (%)	2 (10)	4 (23)	30 (57)	36 (42)
Moderate (8–21 days) (%)	2 (10)	3 (18)	7 (13)	12 (14)
Severe (> 21 days) (%)	1 (5)	0	7 (13)	8 (8)

Group 0 = non-sports group, group 1 = once a week of sports participation, group 2 = at least two times per week of sports participation. NTL; no time loss, i.c. injuries resulted in a participation restriction on the same day of injury complaint but no subsequent restriction, TL (minor, moderate and severe); injuries or illness resulted in restriction of participation of at least 1 day subsequent of the date of injury or illness complaint. ^*a*^Severity of injury or illness; for group 0 it is operationalized as return to physical education, for group 1 and group 2 return to sports

### Injury incidence, characteristics and severity

In total, 46% (*N* = 47) of the participants with CDPD reported 86 injuries during a 12-month follow-up period. Most of the registered injuries (70%) occurred in group 2 (exercising at least twice a week in sports). Almost half of the registered injuries in group 2 resulted in minor (1–7 days) and moderate (8–21 days) time loss in sports participation. While in group 0 and group 1 resp. 89 and 59% of the reported injuries could be classified as NTL, this was different in group 2 with only 40% being classified as NTL. Most of the injuries were articular contusions or distortions (41%), followed by muscles strains (24%), no severe injuries like concussions were reported (Table 3). The majority of the reported injuries were located at the lower extremity (74%). A third of the injuries occurred during organized sports (31%) and only in group 2.

**Table 3:** Type, body location and context in which the injury occurred, categorized per group

Type of injury	Group 0 (Non-sports)	Group 1 (Sports 1x/wk)	Group 2 (Sports ≥2x/wk)	Total (%)
Total number of injuries registered	9	17	60	86
Articular contusion / distortion (%)	3 (33)	4 (24)	28 (47)	35 (41)
Muscles strains (%)	2 (22)	7 (41)	12 (20)	21 (24)
Muscle, tendon (partial) rupture, hematoma / edema (%)	–	1 (6)	5 (8)	6 (7)
Epicondylitis / tendinitis, inflammation (%)	–	3 (17)	4 (7)	7 (9)
Open wound / blister (%)	2 (22)	1 (6)	4 (7)	7 (9)
(sub) luxation (%)	–	–	3 (5)	3 (3)
Fracture (%)	1 (11)	–	2 (3)	3 (3)
Other (chondropathy, ossification) (%)	1 (11)	1 (6)	2 (3)	4 (4)
Body location				
Total number of injuries registered	9	17	60	86
Lower extremity (*n* = 64)				
Hip – upper leg (%)	–	–	11 (18)	11 (13)
Knee – lower leg (%)	4 (44)	7 (41)	15 (25)	26 (30)
Ankle (%)	–	2 (12)	12 (20)	14 (16)
Foot (%)	4 (44)	2 (12)	7 (12)	13 (15)
Upper extremity (*N* = 11)				
Shoulder (%)	–	–	1 (2)	1 (1)
Fingers (%)	–	4 (23)	6 (10)	10 (12)
Spine (*N* = 10)				
Upper (%)	1 (12)	–	5 (8)	6 (7)
Lower (%)	–	2 (12)	2 (3)	4 (5)
Other (face) (*N* = 1)	–	–	1 (2)	1 (1)
Context in which injury occurred				
Total number of injuries registered	9	17	60	86
Organized sports (%)				27 (31)
Team (contact) sports ^a^ (%)	–	–	19 (32)	
Individual sports ^b^ (%)	–	–	8 (13)	
Non-organized sports and play in leisure time ^c^ (%)	6 (67)	3 (18)	14 (23)	23 (27)
Physical education (%)	1 (11)	6 (35)	5 (9)	12 (14)
Activities in daily living (walking, bicycling) (%)	2 (22)	8 (47)	14 (23)	24 (28)

^a^ *Soccer, ice hockey, adapted volleyball, basketball, water polo,* ^*b*^ *road cycling, swimming, athletics, fitness,* ^*c*^ *jumping the trampoline, rowing, sailing, skiing*

### Illness incidence, characteristics and severity

A total of 90 illnesses during a 12-month period were reported by 43 participants (42%) (Table 2). Most of the illnesses were reported in group 2 (59%). Overall, the majority of the illnesses (64%) resulted in a minor time loss (1–7 days) for participation in school, no differences were seen between the three groups. Only 9% of the illnesses were severe (> 21 days). The severity of the illness for return to sports was the highest for group 2 (57%; 1–7 days), compared to group 1 and group 0 for which most of the illnesses resulted in no time loss for return to PE or sports (75 and 59% resp). Flu and fever were the most commonly reported illnesses (58%) followed by symptoms of fatigue (18%). In addition, there were single cases of inflammation, asthma, migraine, epileptic attack, sleeping apnea, shingles and pertussis, which together accounted for 24% of the total reported illnesses.

### Injury and illness rate per 1000 h of PA

The calculated injury rate per 1000 h of PA was for group 0, 1 and 2; 0.84, 1.88 and 1.33 resp. The illness rates per 1000 h of PA was 1.87, 1.88 and 1.18 resp. for participation restriction on the same day of injury or illness complaint but no subsequent restriction. The differences between the three groups were not statistically significant (Tables 4 and 5). The illness rate per 1000 h of PA differed significant between group 2 (*N* = 64) compared to group 0 + group 1 taken together (*N* = 39), *p*-value = 0.028.

**Table 4:** Injury rate per 1000 h of physical activity and comparison of injury rates per group

Group comparisons	Mean minutes PA per day (range min-max)	Cumulative hours of PA during 1 year per group	Number of injuries (95% CI)	Injury rate (95% CI)	IRD (95% CI) IRR (95% CI)	*p*-value*
Group 0 (non-sports) + group 1 (sports 1x/wk) Vs. Group 2 (sports ≥2x/wk)	134.6 (59–244) 171.8 (73–292)	19,693 44,937	26 60 (45.8 to 77.2)	1.32 (0.9 to 1.9) 1.33 (1.0 to 1.7)	IRD −0.015 (−0.6 to 0.6) IRR 0.99 (0.6 to 1.6)	0.962
Group 0 (non-sports) Vs. Group 1 (sports 1x/wk)	146.2 (80–244) 123.6 (59–182)	10,674 9019	9 (4.1 to 17.1) 17 (9.9 to 27.2)	0.84 (0.38 to 1.6) 1.88 (1.1 to 3.1)	IRD 1.04 (−17.1 to 17.7) IRR 0.44 (0.18 to 1.06)	0.275
Group 1 (sports 1x/wk) Vs. Group 2 (sports ≥2x/wk)	123.6 (59–182) 171.8 (73–292)	9019 44,937	17 (9.9 to 27.2) 60 (45.8 to 77.2)	1.88 (1.1 to 3.1) 1.33 (1.0 to 1.7)	IRD 0.55 (−6.3 to 17.2) IRR 0.71 (0.41 to 1.30)	0.387
Group 2 (sports ≥2x/wk) Vs. Group 0 (non-sports)	171.8 (73–292) 146.2 (80–244)	44,937 10,674	60 (45.8 to 77.2) 9 (4.1 to 17.1)	1.33 (1.0 to 1.7) 0.84 (0.38 to 1.6)	IRD 0.49 (−17.6 to 7.2) IRR 1.58 (0.78 to 3.6)	0.295

*PA* physical activity, *CI* confidence interval, *vs*; versus, *IRD* incidence rate difference, *IRR* incidence rate ratio, *significant difference *p*-value ≤0.05

**Table 5:** Illness rate per 1000 h of physical activity and comparison of illness rates per group

Group comparisons	Mean minutes PA per day (range min-max)	Cumulative hours of PA during 1 year per group	Number of illnesses (95% CI)	Illness rate (95% CI)	IRD (95% CI) IRR (95% CI)	*p*-value
Group 0 (non-sports) + group 1 (sports 1x/wk) Vs. Group 2 (sports ≥2x/wk)	134.6 (59–244) 171.8 (73–292)	19,693 44,937	37 (26.1 to 51) 53 (39.7 to 69.3)	1.88 (1.4 to 1.2) 1.18 (1.1 to 1.6)	IRD = 0.7 (0.07 to 1.31) IRR = 1.6 (1.0 to 2.5)	0.028*
Group 0 (non-sports) Vs. Group 1 (sports 1x/wk)	146.2 (80–244) 123.6 (59–182)	10,674 9019	20 (12.2 to 30.9) 17 (9.9 to 27.2)	1.87 (1.9 to 1.3) 1.88 (1.9 to 1.3)	IRD = − 1.12 (− 1.23 to 1.20) IRR = 0.99 (0.5 to 2.0)	0.986
Group 1 (sports 1x/wk) Vs. Group 2 (sports ≥2x/wk)	123.6 (59–182) 171.8 (73–292)	9019 44,937	17 (9.9 to 27.2) 53 (39.7 to 69.3)	1.88 (1.9 to 1.3) 1.18 (1.1 to 1.6)	IRD = 0.7 (−0.1 to 1.5) IRR = 1.59 (0.9 to 2.8)	0.090
Group 2 (sports ≥2x/wk) Vs. Group 0 (non-sports)	171.8 (73–292) 146.2 (80–244)	44,937 10,674	53 (39.7 to 69.3) 20 (12.2 to 30.9)	1.18 (1.1 to 1.6) 1.87 (1.9 to 1.3)	IRD = 0.7 (1.5 to 0.1) IRR =0.63 (0.4 to 1.1)	0.075

*PA* physical activity, *CI* confidence interval, *vs* versus, *IRD* incidence rate difference, *IRR* incidence rate ratio, * significant difference *p*-value ≤0.05

## Discussion

The aim of this study was to investigate the incidence, type, severity and risks of (sports-related) injuries and illnesses among ambulatory youth with CDPD using a cross-sectional analysis of data aggregated and extrapolated over time. We found that participation in sports ≥2 times per week does not pose an increased risk in the incidence of injury or illness per 1000 h of PA in youth with CDPD compared to once weekly or no sports participation.

Children and adolescents with CDPD are increasingly encouraged to participate in sports and exercise [27]. Actual participation minimizes physical inactivity, optimizes physical functioning, promotes inclusion in society and enhances overall well-being in youth with CDPD [3]. Given all these benefits, fear of injury frequently remains a barrier to participate in sports [9]. The question rises whether all the major physical and psychosocial health benefits outweigh the risk of getting injured or ill through sports participation.

To the best of our knowledge this is the first prospective cohort study on identifying and comparing injuries and illnesses among sporting and non-sporting youth with CDPD. We found no significantly evidence that sports participation results in significant higher injury and illness rates among youth with CDPD. A current systematic review reported inconsistent research outcomes about the impact of sports participation on injury rates among healthy youth (age 6 to 15 years) [28]. Some studies concluded that sports participation is the most risky for sustaining injuries, while another study of healthy youth without a CDPD showed that the absolute number of injuries occurring during leisure time and PE are as high as those occurring during sports [28]. The latter is in line with results in our study. The injury rate was not statistically significant between those who participate into sports and those who are not.

As a result of our study, non-sporting participants also get injured, not from sports participation but they get injured by less intense physical activities: PE lessons, ADL or non-organized sports and play in leisure time. Interestingly, youth participating in sport ≥2 times per week were less likely to get injured in daily life situations compared to those who participate in sports once per week. We conjecture that the more frequent sports participant might have better motor skills or participate in low-risk sports, which makes them less vulnerable to an injury in comparison with less frequent sports participants.

### Injury and illness type and severity

Although the absolute number of injuries and illnesses are high in sports participants, the types of injuries and illnesses reported in the current study have no or minimal impact on being able to participate in school, PE or sports. The most reported injuries in our study were contusions/distortions or muscles strains, and located at the lower extremities. Our findings are in line with results of previous studies in both healthy youth and high school athletes with disabilities [14, 29], while in Paralympic athletes most of the injuries were related to overuse (tissue inflammation and pain) [30]. According to time loss by sustaining an injury, our findings are similar with several studies, in which sports injuries suffered by high school athletes with disabilities and Paralympic adult athletes resulted in no or minor loss of training time [15, 29–31]. According to the severity of illnesses, preliminary results in Paralympic adult athletes shows that a higher training load results in a higher number of illnesses (infections) and the type of locomotion seems related to the incidence of illness, i.e. wheelchair athletes reported a high number of upper respiratory tract infections compared to able-bodied athletes [30]. The illness severity in the study of Fahger et al. (2017) was minor, 1–3 days of time loss of training, which is comparable with our study findings, although these studies are not comparable with our research with respect to study design, participants’ age, medical diagnoses and sports level. For instance, the majority of the study population of Ramirez et al. (2008) had a mental disorder like autism and were on average older (18 years), whereas the study population of Fagher et al. (2017) were adult athletes with visual impairments, or were wheelchair dependent. The studies of Blauwet et al. (2016) and Derman et al. (2013) followed the athletes of the London 2012 Paralympic games during 14 days. All these studies, however, show similar results, that the severity of injuries and illnesses are low for peoples’ ADL and/or sports participation. Remarkably, in our study, the incidence illness rate is the lowest in youngsters who participates in sports at least two times per week compared to their peers with less frequent or no sports participation. This result might suggests that regular participation in sports of at least two times a week could have a protective effect against illnesses.

### Strengths and limitations

This study was the first large study to evaluate injuries and illnesses among sporting and non-sporting youth with CDPD over a 12-month period. Strong points are the prospective injury and illness data monitoring in combination with the objective direct measurement of PA by using accelerometry. In addition, the incidence to injuries and illnesses and calculation of rates are based on exposure of objectively measured PA, which is a key factor [32, 33].

Obviously, this study also has some limitations. It could be of interest to conduct analyses per medical diagnose subgroup. Unfortunately, the sample sizes within the different medical diagnose groups in this study were too small for adequate statistical analyses. Moreover, we measured PA during one school week and weekend, assuming that this measurement is a representative week. The injury and illness rate formula assumes that the random sample of 7 days (school week) objectively measured PA distributed over the full study period represents the PA experience of the whole sample and can be used to derive estimates of the population experience over that period. Use of the PA measurements during one school week and extrapolated for a 12-month period is a limitation of the study. Future research should measure PA during a longer period of time and during holidays, were PA levels are probably different from PA during school weeks. Further, the injuries and illnesses were subjectively reported by the parent and/or child and not objectively by a physician or physical therapist as ideally recommended [32]. Participants from all over the Netherlands were enrolled in our study, therefore a physical consultation of injury or illness was not feasible. As a solution, we choose a structured interview by phone to further analyze the injury or illness reported in the online questionnaire. Even so, feasibility of this methodology was time-consuming and required a high degree of precise working, i.e. checking the incoming questionnaires and the presence of an injury or illness. In a pilot study by Fagher et al., the use of a novel eHealth-based application for self-report in Paralympic athletes was generally feasible and usable [30], and seemed less time-consuming compared to our technique. The use of new methods developed for Paralympic athletes may be recommended for use in future recreational-level research to collect this type of data. Moreover, there were more boys compared to girls in our sport-2 group. This may have influenced our results as evidence in healthy youth shows that girls are at increased risk of injuries while participating in PA compared to boys [34]. Low levels of PA and/or physical fitness seemed to increase injury incidence levels, but the exact mechanisms remain unclear [28]. In addition, no information about the pubertal maturation of the participants was collected. During pubertal maturation risk on growth-related injuries is high [34]. Future studies should take aspects like pubertal maturation, training history, training status and physical fitness level into account and may also investigate how the level of sport participation is related to injury and illness risks in order to develop risk profiles and injury prevention programs in more detail for youth with CDPD [30, 32, 34].

## Conclusions

Participation in sports twice a week does not pose an increased risk in the incidence of injury or illness per 1000 h of PA in ambulatory youth with CDPD compared to once or no participation in sports per week. The impact of injuries or illnesses was only minor. Given the evident health benefits for youth with CDPD without obvious risks, sports participation at least twice weekly in youth with CDPD is highly recommended. A next step would be to conduct studies to identify specific variables (i.e. physical fitness, type and training history of sports) that could influence prevention of injuries and illnesses.

## Data Availability

The datasets used and/or analyzed during the current study are available from the corresponding author on reasonable request.

## Abbreviations

ADL: Activities of daily living
ANOVA: Analysis of variance
BMI: Body mass index
CDPD: Chronic disease or physical disability
GMFCS: Gross motor function classification system
MVPA: Moderate-vigorous physical activity
NAIRS: National athletic injury registration system
NTL: Non-time loss
PA: Physical activity
PE: Physical education
SDS: Standard deviation score
STROBE: Strengthening the reporting of observational studies in epidemiology guidelines
TL: Time loss
VPA: Vigorous activity levels
VWS: Dutch ministry of health, welfare and sport
